# An Algorithmic Metaphysics of Self-Patterns

**DOI:** 10.3389/fpsyg.2020.607917

**Published:** 2020-12-04

**Authors:** Majid D. Beni

**Affiliations:** Department of Philosophy, Middle East Technical University, Ankara, Turkey

**Keywords:** coupling, constitution, composition, compressibility, free energy principle, self-pattern

## Abstract

The paper draws on an algorithmic criterion to demonstrate that the self (as initially described in Shaun Gallagher’s a pattern theory of self) is a composite, scattered, and patterned object. It also addresses the question of extendedness of the self-pattern. Based on the criteria drawn from algorithmic complexity, I argue that although the self-pattern possesses a genuinely extended aspect (and in this sense, the self-pattern is minimally extended) the self-pattern and its environment do not constitute a genuine composite object.

## Introduction^1^

This paper does not intend to present a general theory of extendedness. Instead, it focuses on a specific case to address the question of *the relations between the self and its environment* at a fundamental level. The paper endorses the criterion of compressibility-cum-simplicity as the yardstick of the extendedness of *the self*. The general intuition behind this move from the theory of extended cognition to the discussion of extendedness of the self is this: the self is a cognitive agent *par excellence*, and if we unravel the issue of the extendedness of the self adequately (in terms of complexity and simplicity of *patterns*) we will acquire deep insights into the criterion of extendedness of cognition. I conceive of the relationship between the self and its extension in terms of Gallagher’s (2013) “A Pattern Theory of Self” (also see Kyselo, 2014; Beni, 2016; Gallagher and Daly’s, 2018).

Although Gallagher and colleagues speak extensively about the dynamical relations between aspects of the self-pattern (the extended aspect included), they do not address the issue of the ontological state of the patterned self and its aspects. The only exception is their expressed sympathy for Dennett’s (1991) theory of real patterns (Gallagher and Daly’s, 2018, p. 2). The paper considers the self as a composite object and asks two important questions;

1. Is the extended aspect constitutive of the self(-pattern)? If that is the case, there is some purchase for the extendedness of the self under the pattern theory.
2. Do the self-pattern and the environment constitute a genuine composite object?

The notion of “constitution” that is used in the present paper is different from Gallagher’s view of “constitution.” This paper regards “constitution” as a matter of composition, whereas Gallagher (2018) speaks of “dynamical constitution.” “Dynamical constitution” is a term of art, and it could be (and indeed has been) explicated in terms of reciprocal (or circular) causality. Others have defended this view before. Kirchhoff (2015), for example, identifies “constitution” with diachronic causal coupling. Kirchhoff’s view is in harmony with the extended-enactivist approach (as well as with Gallagher’s use of dynamical gestalt). However, I have some reservations about how to construe the “causal coupling” relation in more or less familiar metaphysical terms. I shall unpack this reservation in the remainder of the paper, but for the time being suffice to say that the concern about the causal-coupling notion of constitution is discussed under the coupling-constitutive fallacy (Aizawa, 2010). The fallacy holds that the causal coupling relation is not sufficient for the constitution. And although Gallagher does address the causal-constitution fallacy (Gallagher, 2018), in agreement with the enactivist approach (Kirchhoff, 2015), he eventually renounces the compositional view on “constitution” and eradicates the difference between the notion of “constitution” and “causality” and argues that “dynamical couplings of brain-body-environment constitute the mind” (Gallagher, 2018, p. 208). As I say, I do not engage in a fundamental debate about the plausibility of enactivism. Nor do I claim that Gallagher’s approach simply begs the question of extendedness of the mind. I just claim that his dedication to enactivism and extendedness are not well supported enough. That is to say, despite the remarkable success of enactivism and extendedness in making sense of psychological theories (Varela et al., 1991; Barsalou, 2008; Pezzulo et al., 2012; Bitbol and Gallagher, 2018), these approaches have not been developed into a well-supported metaphysical stance, say, about the objecthood or reality of the extended objects. This does not need to mean that the dynamical (causal-coupling) conception of constitution is generally wanting. Indeed, Gallagher does provide a rather detailed critical discussion of the new mechanist roots of the constitution-coupling notion of constitution (Bitbol and Gallagher, 2018; Gallagher, 2018)^2^. But the problem is that this approach does not expansively elaborate on the ontological aspects of the extended objects. I take a compositional stance on the question of constitution. In defense of this move, I can say that the compositional stance could be developed into a clear metaphysical interpretation of real patterns as well as self-patterns. At the same time, this proposal is unassuming, in the sense that it does not intend to deny the viability of dynamical approaches. Nor does it claim the ultimate superiority of the compositional approach.

Perhaps it was wise, on Gallagher’s part, to take enactivism as a basic perspective whose soundness does not need to be supported by further philosophical argument. But I assume that the compositional view on the constitution (that gives rise to the notorious coupling-constitution fallacy) is as respectable as the rival stance. The present paper pays allegiances to the classical compositional view. It could be granted that a compositional view on the constitution of the self and its relationship with its environment deserves to be heard out too. I pinpoint the need for a compositional approach with a reference to the coupling-constitutive fallacy before going further.

The question of the constitutive/coupling relation between the self and the environment is dictated with an eye to a serious challenge to the theory of extended cognition (Clark and Chalmers, 1998); for the challenge, see Aizawa, 2010; Adams and Aizawa, 2012). I suggest that the criteria of compressibility and simplicity of patterns provide precious insight into the question of extendedness of the self. The proposed criteria are inspired by Steve Petersen’s (Petersen, 2013, 2019) algorithmic metaphysics of the composition of objects (and more originally, by Dennett’s (1991) theory of real patterns, as well as Ladyman and Ross’s (2007) engagement with the idea of real patterns). Dabbling in algorithmic metaphysics in this fashion, I provide answers to the two abovementioned questions. I argue that;

- In answer to (1) above, the extended aspect is constitutive of the self-pattern. The answer is backed up by the criterion of compressibility and its minimal and maximal clauses (as inspired by Petersen’s work). I submit that the self-pattern is a composite object constituted by various aspects.
- In answer to (2) above, I suggest that the self-pattern and the environment do not constitute a composite object. I substantiate this point by invoking the same criteria of compressibility and simplicity. I draw on Friston et al.’s theory of selfhood under the Free Energy Principle to present the criteria of compressibility and simplicity of the self to substantiate my claim.

The paper is structured in the following way. I use a broad brush to sketch some platitudes about the extended cognition thesis as well as the coupling-constitution fallacy. Then I focus on Gallagher’s *a pattern theory* of the self and expose the question of the relationship between various contributors to the self (the cognitive aspect and the extended aspect included). Then I outline Petersen’s patternist criterion of being a composite object and show that the self-pattern is a scattered composite object that subsumes various aspects, elements, and factors as its constituents (the extended aspect included). This provides some purchase for defending a minimal account of the extendedness of the self-pattern (only in the sense that the self has an extended aspect). Then I show that the self-pattern and the environment do not constitute a composite pattern (in an ontologically significant way). The conclusion is that although the self-pattern is minimally extended, the extendedness only amounts to a coupling relation between the self-pattern and its environment but not to a genuine form of constitution.

## The Extended Cognition and the Coupling-Constitution Fallacy

According to the extended cognition thesis, to fulfill our cognitive goals, we depend on elements in our environment, including the “technological gadgets with which we regularly and uncritically interact” (Carter and Kallestrup, 2019, p. 1). The insight into the integration between the cognitive abilities of the organic agents and (presumably) non-organic (or extra-organismic) devices led to the philosophical belief that the extra-organismic devices constitute a non-negligible part of the cognitive process (Clark and Chalmers, 1998). The environmental factors are not only coupled with cognitive processes, they also constitute parts of these cognitive processes. According to Clark and Chalmers (1998, p. 8):

If, as we confront some task, a part of the world functions as a process which, were it done in the head, we would have no hesitation in recognizing as part of the cognitive process, then that part of the world is (so we claim) part of the cognitive process.

Despite its natural appeal, the extended mind thesis has been targeted by the objection from the coupling-constitution fallacy (Adams and Aizawa, 2008, 2009; Aizawa, 2010). The objection is based on a denial: all external resources causally interact with cognitive systems are not integrated with cognitive systems.

The main argument behind the extended mind thesis seems to be something like this: Assume that X is a cognitive process. X is causally dependent on Y and is mutually interacting with it to fulfill its cognitive goals (meaning that Y is not dangling at the end of the causal chain and is included in the loop). It follows that X and Y form an integrated cognitive process. The argument is allegedly based on some fallacy: from the fact that X and Y are causally connected, it does not follow that X and Y form an integrated cognitive process, given that causation simpliciter is not enough for supporting the claim about the constitution of the system (Aizawa, 2010, p. 333). One reason for this pessimism is that “constitution” is considered to be synchronic whereas causality needs to be diachronic. The pessimism could as well be based on some deeper skepticism about the capacity of “causality” to be the universal glue of constitution [As it happens, skepticism about the status of causality finds its way into the work of some notable pattern theorists (Ladyman and Ross, 2007, chapter 5)]. Be that as may, according to Adams and Aizawa (2008, p. 91) “It simply does not follow from the fact that process X is in some way causally connected to a cognitive process that X is thereby part of that cognitive process.”

There are various sorts of reactions to the coupling-constitution fallacy (Wilson, 2004, 2010; Clark, 2008; Rowlands, 2009; Walter and Kyselo, 2009; Piredda, 2017). Advocating an enactivist approach, Gallagher himself considered the coupling-constitution fallacy shortly and let it down rather easily. He asserts that the “diachronic conception of constitution that includes reciprocal causal relations” can be adopted by the enactivist, extended-mind approach, which supports a dynamical and holistic conception of cognition (Gallagher, 2018, p. 207). It is by no means a shortcoming of Gallagher’s approach that it depends on a causal-coupling conception of constitution. This dynamical conception is well-supported enough (Kirchhoff, 2015; Kirchhoff and Kiverstein, 2019). So, instead of trading off intuitions about the (undeniable) appeal of enactivism, I suggest a rather operational (i.e., algorithmic) criterion of testing the metaphysical viability of extendedness. In this respect, the endeavor of this paper is different from the preceding debates of critics and advocates of enactivism and the extended mind theory who do not provide a clear demarcation criterion for distinguishing genuine cases of constitution from cases of coupling simpliciter. Let me elaborate.

The question of extendedness is this: where to draw the boundaries of cognition? The thesis of extendedness indicates that there are no sharp boundaries between the cognitive system and its environment, and the cognitive system and its environment constitute an entity. I submit that the philosophy of selfhood provides a good framework for unraveling the question of extendedness. The self is the cognitive agent par excellence, and the question of extendedness could be rephrased in terms of how to draw the boundaries that separate the self-pattern from its environment. The discussion will be continued in the next section.

## A Pattern Theory of Self

I address the question of extendedness of the self in the context of Gallagher’s (2013) a pattern theory of self (I call it *the* pattern theory but without any specific philosophical intentions). The pattern theory of the self has been discussed expansively (Kyselo, 2014; Newen, 2018; Beni, 2019a,b). While the choice of “the pattern theory” in the context of the present enquiry is to some extent arbitrary, the theory provides a nice venue for pursuing the question of extendedness. This is because the pattern theory stipulates the existence of an extended aspect of the self.

Gallagher states the pattern theory of self in the following manner: “According to the pattern theory, a self is constituted by some characteristic features or aspects that may include minimal embodied, minimal experiential, affective, intersubjective, psychological/cognitive, narrative, *extended*, and *situated* aspects” (Gallagher, 2013, p. 1 emphasis added). According to this approach, what is called the self is a cluster concept that includes a sufficient number of features. Despite speaking of *aspects* of the self, Gallagher endeavors to “stay plural about the concept of self” (Gallagher, 2013, p. 1). If so, the so-called aspects (as being organized into certain patterns according to Gallagher) are not models of something (i.e., the self) that has its independent existence. The point about the existence is rather important in the context of our paper (which is concerned with *metaphysical* issues).

The self is not a simple entity with its independent existence. However, it is not obvious that the self-pattern does not exist at all (I will follow Gallagher, 2013 and use “self-pattern” and “self” interchangeably). The pattern theory does not advocate a form of eliminativism about the self (Metzinger, 2003). The self-pattern is not non-existent in the context of the theory. What manner of existence does the self-pattern possess then? From the metaphysical point of view, we can assume that the self-pattern (which is neither independently existent nor totally non-existent) exists as a composite object, constituted by the menagerie of various aspects and elements. The self-pattern and its aspects and elements do not exist independently of one another. In this paper, I argue that the elements and aspects of the self are constitutive of the self-pattern, which is a scattered composite object.

To a first approximation, Gallagher’s definition of “self-pattern” does not provide a clear insight into the ontological status of the self. Gallagher suggests that “what we call self consists of a complex and sufficient pattern of certain contributors, none of which on their own is necessary or essential to any particular self” (Gallagher, 2013, p. 3). What is the relation between contributors of the self? The pattern theory emphasizes the diversity of aspects and elements of the self. However, it does not account for the relation between aspects quite sufficiently, meaning that it offers “no account of the individual as explanatory whole” (Kyselo, 2014, p. 1). In other words, despite acknowledging the existence of meaningful dynamical relations between self-patterns, Gallagher’s account “doesn’t develop a full theory about how the various elements of the pattern of self are connected” (Beni, 2016, p. 3,731). Although these objections are directed at the pattern theory in the first place, they also bear on the issue of the extendedness of the self. To support an extended conception of the self we need to accept that the self always latches onto the ecological and/or social environment, and the unit of analysis is the self-environment (Gallagher, 2013, p. 4). But if the pattern theory fails to produce a full account of the relationship between different aspects of the self, trivially it would fail to explain how extended (and situated) contributors are indeed component parts of the self-pattern in a constitutive sense. Accordingly, the pattern theory would fail to account for the extendedness of the self-pattern. This could be a significant blow to the extended cognition thesis as well as a general objection to the pattern theory. That said, I have to immediately add that Gallagher and Daly offer some interesting strategies for accounting for the relationship between diverse aspects of the self. The most promising of their suggested strategies (in my opinion) consists of invoking predictive processing and the free energy principle. Why is this the case? Patterns that are at issue in the pattern theory are specified in terms of dynamical system theory. Gallagher’s insight into that subject receives support from some important works such as (Schöner and Kelso, 1988; Kelso, 2016). However, this paper assumes that it could be *also* worthwhile to invoke comprehensive and unifying formal framework under which to model relations between diverse aspects of the self (as well as the relation between the self and the environment). The Free Energy Principle (FEP) seems to underpin such a comprehensive, unifying framework. The dynamic approach too endeavors to account for the emergence of the patterned behavior under generative self-organizing processes (Schöner and Kelso, 1988; Kelso, 2016). FEP can be used in the same spirit to achieve the same goal with remarkable formal precision and empirical success. In view of the mathematical vigor and empirical success of the FEP, it seems that FEP provides a suitable theoretical framework for bolstering Gallagher’s account of the relationship between aspects of self-patterns.

The Free Energy Principle (FEP) and predictive processing, characterized in terms of Bayesian models of minimization of variational free energy, are the unifying theoretical framework that accounts for perception, cognition, and action (Friston, 2010; Hohwy, 2013; Clark, 2016). In order to survive, organisms must remain in *non-equilibrium steady states*. This means that they must avoid getting into unpredicted situations. The probabilistic description of the dynamics of systems in non-equilibrium steady states is developed into two kinds of descriptions. According to Ramstead et al. (2020, p. 6):

First, the system can be described in terms of the flow of the system’s states—that are subject to random fluctuations—in which case, we can formulate the flow in terms of a path integral formulation, as a path of least action. Equivalently, we can describe the non-equilibrium steady-state in terms of the probability of finding the system in some state when sampling at any random time.

According to this formulation, self-organizing systems (in terms of intrinsic geometry) evolve toward some non-equilibrium steady-state density which can be interpreted as a statistical or generative model (in terms of its extrinsic geometry). In this fashion, we could characterize the joint probability density over internal states and external states (Ramstead et al., 2020, p. 9). Within this context, variational free energy is an information-theoretic measure that provides an upper bound on surprise. Entropy is “[t]he average surprise of outcomes sampled from a probability distribution or density” (Friston, 2010, p. 1). Living systems minimize their free energy by staying in a small set of environmental states. A fish needs to stay in the water because a fish out of water will find itself in a surprising state. Staying in a limited number of states enables organisms to form approximately precise predictions of the environment. This makes the organisms’ interactions with the environment efficient. The organism can minimize its free energy either via adjusting its models (that’s predictive coding) or via action (that’s active inference). It can minimize its free energy by either changing its internal models of the environment based on evidence that is sampled actively or by acting on the environment and changing the environmental states to make them match its predictions. When applied to the brain, the theory holds that the brain could get approximate representations of the causal structure of the environment by minimizing prediction errors^3^. Below, I shall unpack this remark.

The brain forms generative models^4^ of the environment and through top-down processing in a hierarchical organization represents the real world. In case of discrepancy between predictions and actual sensory inputs, the brain minimizes its prediction errors and finesses its generative models (or the organism changes the environmental states to match the predictions) (Friston and Stephan, 2007). FEP and predictive processing are used to provide viable models of selfhood (Limanowski and Blankenburg, 2013; Apps and Tsakiris, 2014; Limanowski and Friston, 2020). At least for some organisms, having a representation of the self in generative models is indispensable to the multisensory integration in both exteroceptive and interoceptive streams. On such grounds, Gallagher and Daly’s (2018, p. 8) argue that FEP and predictive processing characterize the dynamical relations that bring together otherwise diverse self-patterns. Let us see how this affects the extendedness of the self.

Because there are dynamical relations between self-patterns, it can be assumed that the extended aspect is somewhat connected to other aspects of the self. But does this mean that the extended aspect is a *constituent* of the self (in contrast, it could be assumed that it is related to other self-aspects loosely and without forging any strong ontological bonds? Gallagher and Daly’s elaboration on dynamical relations between self-patterns is silent about this. Moreover, aside from a fleeting reference to Dennett’s (1991) theory of real patterns, Gallagher and Daly do not explicate their view on the existence of the self-pattern. The question of (modes of) the existence and reality of the self needs to be treated with adequate technical tools.

Gallagher and Daly’s characterization of dynamical relations between aspects of the self indicates that Gallagher is not committed to the existence of a class of totally diversified and disintegrated self-contributors. Nor does he conceive of the self-pattern in terms of a classical substance. This puts the ontological status of the self-pattern in a twilight zoon. Inspired by Gallagher and Daly’s, (2018 p. 2) remark on the Dennettian tendency of their view, I suggest that the self is a scattered composite pattern that is constituted by diverse aspects, the extended aspect included. I use metaphysical tools that are congenial to Dennett’s (1991) theory of *real patterns* to substantiate my stance on the existence and reality of the self as a composite pattern. It is true that at times Dennett seems something of a pragmatist about the reality of the pattern, and doesn’t offer any heavy ontology^5^. However, Dennett (1983, p. 380) is clear that he is not a fictionalist about theoretical posits such as the center of gravity. This is because these posits play an explanatory function (and thus could be embraced based on some indispensability argument). The result is a moderate metaphysical stance that cannot be described in simple terms of realism vs. instrumentalism. According to Dennett, his real patterns theory “is clearer than either of the labels [meaning realism and instrumentalism],” so he just leaves “that question to anyone who still finds illumination in them” (Dennett, 1991, p. 51). This approach is in harmony with Petersen’s take on algorithmic metaphysics. Petersen submits that “I must confess that I am sympathetic not only to Dennett’s patternist proposal, but also to this metaontological stance [of Dennett’s, which has been just cited]” (Petersen, 2019, p. 3). I suggest that this metaphysical enterprise can be applied to deal with the question of the extendedness of the self-pattern.

More light will be shed on this topic if we ponder the two following questions:

1. Is the extended aspect constitutive of the self-pattern?
2. Do the self-pattern and the environment constitute a genuine composite object?

We need to know more about the metaphysics of composed patterns before providing viable answers to these questions.

## An Algorithmic Metaphysics of Composition

We can address the question of how to draw the boundaries of a cognitive system if we could tell when two systems that are coupled form an integrated system. This question resembles the question of *composition*, which asks when we can claim that some objects constitute a new object. This paper takes a compositional stance on constitution.

Generally, the question of the composition provides metaphysical insights into the thesis of extendedness. It may be assumed that there are no composite objects at all, or it may be assumed that any mereological sum constitutes an integrated object. Between these two extremes, there are moderate varieties; some pluralities (such as atoms of hydrogen and oxygen) constitute a new object (such as a molecule of water) and some other pluralities (such as the compound of the pear tree in my yard and the Taj Mahal) do not constitute a new object^6^. In this context, Petersen is advocating a compositional conception of constitution (Petersen, 2013, p. 312). According to this approach, for an object to be *constituted*/*composed* by some pluralities, there must exist some degree of “connectedness” or “integrity” between the pluralities (Simons, 2000, p. 290). Integrity and connectedness are conditions that need to be satisfied by constitution. This is because without integrity and connectedness the aggregates would be assembled into an arbitrary sum. In this sense, I adopt a compositional stance on constitution (Mark the similarity of the problem of composition/constitution to the problem of the relation between self-contributors and aspects. The general insight of this paper is that from a metaphysical point of view, the self can be identified with a scattered composite pattern).

There have been significant attempts at invoking information-theoretic frameworks for identifying the structure of reality, or more technically, real patterns (Dennett, 1991; Ross, 2000; Ladyman and Ross, 2007). According to Dennett’s statement of the patternist approach, “A pattern exists in some data-is-real-if there is a description of the data that is more efficient than the bit map, whether or not anyone can concoct it” (Dennett, 1991, p. 34). Interestingly enough, Dennett’s conception of real patterns is in line with Gallagher and Daly’s conception of self-patterns [Referring to Dennett’s pattern theory, Gallagher indicates that “the self has the scientifically useful reality of a pattern” (Gallagher and Daly’s, 2018, p. 2)]. I shall flesh out this proposal with an eye to its use for dealing with the question of the self (as a composite reality) and its metaphysical aspects. This proposal draws a connection between the metaphysical definition of objects (as patterned or structured entities) and their description in terms of compressibility (compressible objects are patterned). Patterns that are described by compressed programs are indispensable to viable representations of the world. Dennett generally implies that the patterns could be characterized based on Kolmogorov complexity. James Ladyman and Don Ross have used the ideas of logical depth^7^ and projectibility to characterize the patterns. Projectible patterns, say Ladyman and Ross, are real patterns in the dataset^8^,^9^. Petersen (2013, 2019) characterizes real patterns by invoking algorithmic information theory (and more specifically, in terms of Kolmogorov complexity).

Petersen’s goal is to show how the patternist approach can make sense of “composition” which is metaphysically a vague and mysterious notion. Kolmogorov complexity [K (x)] efficiently represents the main insight behind Dennett’s and Ladyman and Ross’s views on the representation of reality. Here, the notion of incompressibility (in terms of Kolmogorov complexity or logical depth, which are arguably translatable to one another) provides a criterion of the constitution of an object, given that “To be is to be a real pattern” (Ladyman and Ross, 2007, p. 233). The relevance of the present discussion to the issue of extendedness is this: the criterion of being a real composite could be used to determine whether the biological cognitive system and the environment form a real composite entity. More specifically, I argue that the criterion can be used to make sense of the integrity and connectedness of the extended aspect with the rest of the self-pattern. We need to show that the extended aspect is constitutive of the self. Then we can conclude that the self-pattern is minimally extended. Below, I shall furnish more details about the criterion of being a composite patterned object.

According to Petersen (2013, 2019) the criterion of being a real pattern (characterized in terms of Kolmogorov complexity) can demarcate what is a genuine composite object from the mere sum of independent objects or patterns. According to Petersen’s proposal, an aggregate of objects is itself a real object if there is some kind of integrity and connectedness between its component parts. In other words, real composite objects are simpler than the sum of their independent component parts. In this fashion, Kolmogorov complexity can be incorporated into an ontological criterion of what is real. According to Petersen, given that “compressibility” corresponds to “simplicity,” there is ontological gain when there is some gain in a pattern. This definition provides insights into the internal integrity of genuine composite objects. This is because “to compose, a compressible region must be referenced by the best compression of the totality in which the region resides” (Petersen, 2019, p. 10). I unfold the technical details immediately.

Complexity and simplicity are defined in terms of the processing of information in a universal Turing machine, which is an abstract device that can model any computable algorithm in a discrete domain. A Turing machine is constituted by a finite program. It can manipulate a tape (which is a linear list of cells), and it has a head. The machine can fill each cell with any of the symbols from a specified set of variables, and it can move the head to any specific cell. Based on such simple operations, a Turing machine can model everything in the discrete domain that is intuitively computable. A universal Turing machine can model the behavior of any other Turing machine (Vitanyi, 2009). The relation between the notions of “Turing computation” and “Kolmogorov complexity” is this: Kolmogorov complexity of an object consists of the length of the shortest program (i.e., shortest input) that produces that object, assuming that the program is processed by a fixed universal prefix Turing machine [not all theorists agree that the domain of computable should be discrete (Hutter, 2008)]. The Turing machine program is the description of that object, and an object that has such a shortest description is considered to be simple. Technically, for the string x, the program p provides the shortest description, if when processed by the universal Turing machine U P outputs x. Under that supposition, the shortest description is provided by

K(x)U:=min{l(p):U(p)=x}p

l(*p*) submits the length of p in bits (Hutter, 2008). The definition of complexity that is at issue here is compatible with the definition of logical depth as stated above. And Petersen builds upon the formal definition of Kolmogorov complexity to address the metaphysical question of objecthood in a world that includes some fundamental objects and simple properties (this world also accommodates the succession of time). The question is this: could there be composite objects in this world. This leads us to another important question: what is the criterion of demarcating genuine composite objects from compounds that do not constitute genuine objects. To find answers to these questions, Petersen develops an algorithmic-compositional concept of “constitution” for both objects and their properties (Petersen, 2013, p. 312). I cite Petersen (2013, pp. 308–309) to show how Kolmogorov complexity is developed into a criterion of being a composite object. Let *l ∈ L* be any interval and *x*_*l*_ be a composite function restricted to that interval. *x^#^_*l*_* designates the length of *x* plus some small constant that denotes the computational overhead;

*x_*l*_ is a* ***composite object*** *if and only if*
*1. KU* (*x*_*l*_) *< x^#^_*l*_ (the* ***compressible clause***).
*2. There is no partition of l into intervals {l*_1_.*.. l_*n*_} such that ∑_*i*_*KU*(*xli*)≤*KU*(*xl*) (the* ***minimal clause***).
*3. There is no interval l’ containing l such that KU*(*xl’*) *≤* K*U*(*xl*) *(the* ***maximal clause****).*

Minimal clause indicates that If x has a sub-region that does not contribute to the compressibility of the remainder, then the diverse components in x do not constitute anything (Petersen, 2019, p. 13). Consider two objects (such as the pear tree and the Taj Mahal, call them o_1_ and o_2_) that do not constitute a genuine composite object (call it o_3_ which is equal to o_1_ ∪ o_2_). As Petersen argues, although o_3_ is compressible, it is not an object because of the minimal clause, given that *KU(o_1_)+KU(o_2_) ≤ KU(o_3_)* (Petersen, 2013, pp. 309–310). Thus, the union of the pear tree and the Taj Mahal is not a genuine object. On the other hand, maximal clause indicates that “parts must each be simpler than wholes, but wholes must be simpler than all their parts taken separately” (Petersen, 2019, p. 15). Consider the possibility of breaking the program that describes *o*_1_ into two substrings *o*_1R_ and *o*_1L_ (representing the right and left substrings). That is to say, o_1_ = o_1R_ U o_1L_. It might be assumed that the same kind of argument that was mentioned to rule out o_3_ as a genuine object could be used to rule out o_1_ too, by indicating that it runs afoul of the minimal clause (instead *o*_1R_ and *o*_1L_ are genuine objects). The branches and the trunk of the same pear tree (or its atoms) could be modeled as separate objects, and it could be assumed that the pear tree itself is not a genuine object. The maximal clause excludes this option by preventing the arbitrary division of proper objects. That is to say, *o*_1R_ and *o*_1L_ are not proper objects. Although in principle we may be able to decompose the pear tree into the independent classes of its branches and its trunk, there is no gain in simplicity or ontology of decomposing the tree in this fashion. Let us see how this applies to the question of constitution of the self and its extendedness.

## Is the Self a Composite Object?

Petersen (2019, p. 5) submits that “Seeking to minimize Bayesian surprise on higher-order parameters is basically just pattern extraction.” FEP is stated in terms of Shannon information theory (rather than Kolmogorov complexity). But there are formal links between Shannon information theory and Kolmogorov complexity (Grunwald and Vitanyi, 2004). At any rate, FEP and predictive processing are used to characterize the self. The self is a composite pattern. It is compressible in sense of Kolmogorov complexity and logical depth.

According to Gallagher, the self does not have an independent existence. But from the point of view of algorithmic metaphysics, the question is not about *the independent existence* of the self but the composition of the self. This is in line with a compositional metaphysical view. The question that we must attend to is this: *Is the self-pattern simpler or more compressible than the sum of its independent contributors*. A positive answer to this question indicates that aspects are constituting the self, instead of loosely hanging together, and it would follow that the self has a genuinely extended aspect. This follows from the application of the metaphysical criterion of compositionality. Below, I explain how these three clauses apply to the issue of extendedness of the self.

As to the first clause, it could be easily granted that the self-pattern is compressible. But does this mean that the self is a composite pattern? According to the minimal clause, if the self is a genuine composite object, the sum of independent aspects of the self cannot be simpler (or more compressible) than the self. It could be the case that the extended aspect and the cognitive aspect are each simpler than the self as a composite object, but the sum of all involved self-contributors is not simpler or less complex than the self-pattern (see the maximal clause in the previous section). Together, the minimal and maximal clauses indicate that for the self to be a genuine composite object (or pattern), its description must be simpler and shorter than the sum of descriptions of diverse self-aspects and contributors. There is nothing in the definition of self-patterns that preclude this possibility. Take Newen et al.’s conception of patterns, which is adopted by Gallagher (2013):

A feature F is constitutive for a pattern X if it is part of at least one set of features which is minimally sufficient for a token to belong to a type X. “Minimally sufficient” means that these features are jointly sufficient for the episode to be of type X, but if one of them were taken away the episode would no longer count as an instance of X (Newen et al., 2015, p. 195).

This definition does not indicate that separate aspects have their independent existent, or the sum of independent self-contributors is more endurable, compressible, or simpler than the self as a composite pattern. That is to say, although the self does not have an independent existence, self-aspects are even less capable of having their independent existence. In this sense, it could be assumed that self-aspects are constituting the self, and the self-pattern is ontologically more fundamental than separate self-aspects. This provides an insight into the constitution of the self. A more technical demonstration can be offered in terms of the FEP-based characterization of the self.

FEP is indeed formulated in terms of Shannon information theory, rather than Kolmogorov complexity^10^. Even so, the FEP-based account considers the self as a theoretical posit that can reduce the complexity of our explanation of various aspects and elements. The sum of separate self-aspects cannot explain cognition and action of a person in a simple and unified way. This means that the sum of explanations that diverse self-aspects produce is more complex than their integrated explanation under the rubric of FEP. The general insight here is that the self is formed around the idea that “one’s own body is the one which has the highest probability of being ‘me’ as other objects are probabilistically less likely to evoke the same sensory inputs” (Apps and Tsakiris, 2014, p. 6). Therefore, stipulating the self as a theoretical posit maximizes the simplicity of cognitive and biological mechanisms by minimizing the overall information conveyed in the system (that is the entropy^11^ of the system that represent the distribution of probabilities that represent the structure of the environment). In words of Apps and Tsakiris, “the notion that there is a ‘self’ is the most parsimonious and accurate explanation for sensory inputs. In mathematical terms, this parsimonious accuracy is exactly the quantity that is optimized when minimizing free energy or prediction error” (Apps and Tsakiris, 2014, p. 89). Gallagher and Daly build upon this fundamental insight to substantiate their view on the existence of meaningful dynamical relations between diverse self-aspects^12^.

A high-level description of the self as a unified entity can explain how minimizing the discrepancy between the generative models and the environment (and one’s own body) generates perception and cognition. The sum of independent self-aspects fails to explain the organism’s representational and active capacities with the same amount of simplicity and fruitfulness. If that is true, then the self is more than just a cluster concept (as Gallagher’s original pattern theory in 2013 paper indicates). Self indeed lacks an independent existence, but it contributes to simpler explanatory schemes in ways that remain beyond the sum of diverse self-aspects.

Finally, it is worth mentioning that Petersen’s definition of composite objects allows for the existence of scattered objects. For example, it indicates that although there are no strong bonds between water molecules that constitute a cloud, the cloud can be recognized as a composite object, albeit a scattered one (Petersen, 2013, p. 311, 2019, p. 8). In this fashion, the self can be identified as a scattered composite pattern. This is because stipulating the self leads to a less complex description of the organization of multiple self-aspects. I shall unfold the consequences for the extendedness of the self.

From our discussion in this section, it follows that the extended aspect is not just loosely hanged to the aggregate of other independent self-aspects. The extended aspect, along with other elements, constitute the self. This means that the self is *minimally* extended. The extended aspect is not just *coupled* with other aspects, they *constitute* a genuinely composite entity, in the sense that is at issue in the compositional view on constitution.

It is worth repeating that Gallagher takes an enactivist stance on the question of constitution, and explicates it in terms of “reciprocal causal relations” (Gallagher, 2018). Accordingly, Gallagher ignores the coupling-constitution fallacy and takes the viability of the extended mind approach for granted. While I do not challenge the validity of the enactivist stance, I do not think philosophical fundamental stances would be justified, confirmed, or verified easily. One can embrace them by pondering a number of various considerations, such as simplicity, fruitfulness, etc. While I do not challenge the general plausibility of the enactivist stance, but I think the compositional view deserves to be taken seriously too. Gallagher’s theory does indicate that the self includes an extended embodied aspect, albeit without appealing to a compositional criterion of constitution. It might indeed be possible to understand the cluster concept of the self (which also embeds an extended aspect) in terms of a dynamical gestalt, constituted by reciprocal causal relations (and thereby by a coupling relation with the environment) rather than compositionality)^13^. This paper does not aim to refute the enactivist approach. It only aspires to provide a metaphysically well-posed alternative to it. This is stated in terms of a criterion of compositionality, and it has the edge over the dynamical systems approach in the following way: the dynamical system approach cannot set a meaningful distinction between causally related clusters that do constitute an object (such as the self) from causal-coupling relations that do not constitute an object (such as the compound of the self and the environment). The compositional approach can set such a distinction. I understand that the advantage that I attribute to the compositional approach may not persuade the enactivist to embrace my proposal. I simply state the compositional criterion to argue that the self and the environment constitute a composite system, without claiming the absolute superiority of this construal over enactivism (the paper is rather unassuming in this sense). In the next section, I will consider the question of the extendedness of the self by asking whether the “self-environment” is a genuine composite entity.

## Do the Self and the Environment Constitute an Object?

As we have already seen, the self can be characterized in terms of FEP and predictive processing. There are ecological and enactivist construals of predictive processing and active inference (Bruineberg et al., 2016; Gallagher and Allen, 2016). Predictive processing and FEP are concerned with the state of homeostasis, which is the state of stable internal equilibrium of the organism with the environment. The ecological construal of FEP and predictive processing represent the relation between the organism and its environment in terms of dynamical coupling of the organism with the eco-niches and its windows of affordance. There are also ecological and enactivist theories of the self and “mineness” in terms of active inference under FEP (Kiverstein, 2018). The question of extendedness of the self is this: Do the self and the environment constitute a genuine composite object, or they are just coupled together? The point that “[t]he organism embodies in its biological organization a hierarchically structured model of its *own* existence in its environment, or equivalently its being-in-the-world” (Kiverstein, 2018, p. 2) could be appreciated rather easily in the context of the pattern theory of the self. This is because (according to what we saw in the previous section) the self-pattern includes an extended aspect. But this is not quite enough for establishing the point that the self and the environment are component of a genuine composite object. I shall clarify immediately.

Self-organizing systems are minimizing their free energy by garnering evidence for their inbuilt generative models. They are self-evidencing in the sense that they endeavor to actively garner evidence for their existence (Hohwy, 2014). And some of these self-evidencing organisms are specified as “selves” or as “subjects of minimal phenomenal experiences” under FEP (Limanowski and Blankenburg, 2013; Kiverstein, 2018). One way of fleshing out this is by assuming that FEP can be used to describe the self as a subject of phenomenal experience. Selves are capable of modeling their *expectations* about the *future* states and the *consequences* of their actions (Friston, 2018, p. 579). Selves (as subjects) can model different consequences of their actions for themselves and choose one particular course of action amongst several possible ones (Friston, 2018, p. 6). In other words, we need to have models of ourselves as trajectories with non-linear effects on our sensory input. This accounts for perceptual unity in a wide time-perspective (Hohwy, 2013, chapter 10). According to Hohwy:

Action arises when prediction error minimization happens by acting on the world while sticking with one’s counterfactual about the world. For this kind of strategy to be feasible we need an ordering of policies for how to go about minimizing error in this way. Such policies are expectations about how flows of error are minimized as we move through the world. These expectations must rely on hypotheses under a hierarchical model of ourselves including our own mental states as coherent and unitary causal trajectories (Hohwy, 2013, p. 255).

In this vein, a self-conscious system is defined as “a system that can simulate multiple futures, under different actions, and select the action that has the least surprising outcome” (Friston, 2018, p. 5). But does this mean that the self is a component of a genuine composite entity (call it the self-environment compound), in a way that is demanded by a strong version of the extended thesis?

A strong version of the extended thesis can be stated like this: the self is extended to the environment, and the self-environment compound is constituted by both the environment and the self as its constituents. If so, the existence of the self depends on its role as a constituent of the self-environment compound. To substantiate this claim within the patternist framework we must be able to show that the self-environment compound is simpler or more compressible than the sum of the self and the environment as independent entities.

Let us grant that the self-environment compound is compressible (this means that we can grant the compressible clause). However, it is not the case that the self-environment compound is simpler or has a more independent existence than the sum of independent components—namely the self and the environment. I shall unfold this remark immediately.

The self and its aspects are described via Markovian models (Friston et al., 2020; Parr et al., 2020). Markov blankets are networks in Bayesian spaces that register a separation between sensory states and active ones, given that sensory states are independent of internal states and active states are independent of external states. Friston et al. (2020) weaved Markovian blankets into a framework of information geometry. The result is an informational/probabilistic description of the way that the brain represents the external world to itself based on the relationship between “probability distribution *about* things” and “probability distribution *of* things” (Friston et al., 2020). Their description of the brain-world relationship accommodates representation of *expected surprise* as the set of beliefs that organisms hold about the consequences of their actions in the world (this provides a basis for phenomenal aspects of the minimal self). Not only Markovian models (with separable internal and external spaces) describe the relationship between the brain and the world, they also model notions of agency, consciousness, and deliberate pre-meditated action (as the properties of the minimal self).

To return to the discussion of compressibility (and minimal and maximal clauses), when constructing their models of consciousness and agency Friston et al. (2020), employed an information geometry that includes a metric for measuring the distance to informational states space. Why this is relevant to the issue of compressibility? Because the information-theoretic measure provides a formal criterion for dealing with the question of compressibility and simplicity. To explicate compressibility and simplicity in information geometry, we should consider the following question. Which of the two classes of entities is simpler or more compressible? The self-environment compound or the sum of the self and the environment as separate entities? Not only the self-environment as a composite entity is less simple than the self and the environment (and their sum), the formal statement of the self and its phenomenal aspects indicates that they are not constituents of the self-environment compound (in the compositional sense). Using Markovian models indicates that to be modeled, the self, as a self-evidencing organism, must be described as an entity with rather clear boundaries that separate it from its environment.

On the same subject, an advocate of the enactivist, extended mind approach does not need to assume that Markov blankets are only in the business of separating inside from outside. Markov blankets can be also used to show how inside and outside are connected (or coupled). Once more, the disagreement about the issue of “constitution” and its relation with “causal coupling” raises its head. For the enactivist, who assumes that causal coupling is enough for the constitution, Markovian models are venues of extension of cognition (and selfhood). However, for those who advocate the compositional view of constitution, the coupling relation is not enough for establishing the extendedness of the self.

The significance of the barrier or the evidentiary boundary between the self and the environment has been emphasized by Hohwy (2007, 2013). The point about the use of Markovian models in describing the brain-world relationship cements the importance of the barrier between the self with its environment (Hohwy, 2017; Kirchhoff et al., 2018). It is possible to see Markov blankets as the venue of dynamical interaction between the organism and its environment. Even so, there is a solid construal which represents Markov blankets as separating boundaries that seclude the organism (or its *self*) from the environment. Although the self is not completely secluded from the environment by boundaries of skin and skull (Kirchhoff et al., 2018; Kiverstein and Rietveld, 2018), the use of the Markov blanket implies that there are staunch boundaries between the self and its environment. This is in line with Hohwy’s (2013, 2014, 2017) representationalist construal of FEP. According to this construal, the brain is secluded from the world, and it infers the state of the world from beyond an inferential veil. The Markov blanket here sets robust boundaries that separate the self from its environment. Aside from Hohwy’s construal, Friston and colleagues have suggested that the Markov blanket could contribute to separating boundaries (Friston et al., 2020; Parr et al., 2020). This latter construal (which presents the Markov blanket as a dividing boundary) is in harmony with assuming that the self and the environment do not constitute a non-decomposable composite entity.

## Concluding Remarks

The paper invoked the criteria of simplicity and complexity of real patterns to deal with two specific questions.

1. Is the extended aspect constitutive of the self-pattern? If this is the case, there is some purchase for the extendedness of the self under the pattern theory.
2. Do the self and the environment constitute a genuine composite object?

Applying the criteria that are drawn from Petersen’s algorithmic metaphysics, I argued that while there is some basic purchase for arguing that the self is minimally extended, the self and the environment do not constitute a real composite object.

## Data Availability Statement

The original contributions presented in the study are included in the article/supplementary material, further inquiries can be directed to the corresponding author/s.

## Author Contributions

The author confirms being the sole contributor of this work and has approved it for publication.

## Conflict of Interest

The authors declare that the research was conducted in the absence of any commercial or financial relationships that could be construed as a potential conflict of interest.

## Footnotes

1 The paper has benefited a lot from the contribution of two referees of this journal, the editor, and steve elliot, these debts are gratefully acknowledged.

2 I owe this important remark to one of the reviewers of this paper.

3 Not all representatives of predictive processing would agree to using “representations” in this context. A radical embodied approach would deny that internal models or inner simulacrums play a significant role in PP. But moderate advocates of emboidement such as Clark conceds that models (which embed representations) do not need to be totally eliminated from predictive processing. According to Clark’s moderate version of embodiment, “it is surely that very model-invoking schema that allows us to understand how it is that these looping dynamical regimes arise and enable such spectacular results” (Clark, 2016, p. 293).

4 Generative models are internal probabilistic models that the brain uses to update its posterior models.

5 I thank one of the reviewers of this journal for reminding me of this point.

6 To the question composition, van Inwagen provides a simple answer in terms of organicism, which holds that “the activity of the xs constitutes a life or the xs are the current objects of a history of maintenance” (van Inwagen, 1995, p. 138). Xs that constitute a life do compose exactly an organism (ibid, p. 91). Of course, the organicist criterion of composition can lead to a rough and ready answer to the question of how to draw the boundaries of cognitive systems—obviously by laying the boundaries of cognition (or composition of the object) on the boundaries of the organism’s body. But for one thing, the criterion precludes the possibility of extended cognition into non-organismic objects too trivially (perhaps based on an unsubstantiated prejudice in favor of being organic). Moreover, organicism may be construed to indicate that only living organisms and mereological simples exist, but there are no non-living composite objects such as tables and chairs. This view, called “the denial” by van Inwagen (1995, p. 1) is too radical to be justified easily.

7 Logical depth is defined as “a normalized quantitative index of the execution time required to generate the model of the real pattern in question by a near incompressible universal computer program, that is, one not itself computable as the output of a significantly more concise program” (Ladyman and Ross, 2007, p. 220).

8 According to this proposal: To be is to be a real pattern; and a pattern x → y is real iff

(i) it is projectible; and
(ii) it has a model that carries information about at least one pattern P in an encoding that has a logical depth less than the bitmap encoding of P, and where P is not projectible by a physically possible device computing information about another real pattern of lower logical depth than x → y (Ladyman and Ross, 2007, p. 233).

9 It has been contended that this criterion of projectibility cannot demarcate *real* patterns (or at least partial non-redundant patterns) from patterns simpliciter (Beni, 2017; Suñé and Martínez, 2019). But these considerations do not deter us from continuing our pursuit, because our present enquiry is not concerned with the association between non-compressibility and *reality* (more on this later in the paper).

10 The difference between Shannon theory of information (which provides the theoretical foundation of FEP) and Kolmogorov complexity is that the former models the randomness of the source of information whereas the latter describes the randomness of the object itself (Grunwald and Vitanyi, 2004, p. 3).

11 Formally, entropy is defined in terms of the amount of information that an observer would gain after receiving a given message. For a random variable X, Shannon entropy is defined as:

$$H\,X=\sum_{x\in X}p\,x\,\log1/p\,x$$

12 Once more, please note that because FEP and predictive processing are stated in terms of Shannon information theory, they are not concerned with the complexity of the object (so much as the source of information). However, FEP conveys clear implications about the simplicity that the assumption of the existence of the self brings to the explanation of cognition and action (not the same could be told of diverse self-patterns).

13 I thank one of the reviewers of this journal for pointing out this to me.
